# Impact of Treatment Delay and Prognostic Factors in Acute Myelitis of Neuromyelitis Optica Spectrum Disease

**DOI:** 10.1002/brb3.70472

**Published:** 2025-04-07

**Authors:** Luyao Zhou, Ziyu Liao, Yingming Long, Zhibin Li, Wei Qiu, Zefeng Tan, Tingting Lu

**Affiliations:** ^1^ Department of Neurology The Third Affiliated Hospital of Sun Yat‐sen University Guangzhou China; ^2^ Department of Radiology Daping Hospital, Army Medical University Chongqing China; ^3^ Department of Neurology The First People's Hospital of Foshan Foshan China

**Keywords:** myelitis, neuromyelitis optica spectrum disease, poor prognosis, treatment delay

## Abstract

**Objective:**

This study aims to investigate the relationship between treatment delay and poor prognosis in acute myelitis of Neuromyelitis Optica Spectrum Disorder (NMOSD). Additionally, we seek to explore the related factors that contribute to poor prognosis, with the intention of providing more precise clinical guidance.

**Methods:**

We retrospectively analyzed the acute myelitis attacks of NMOSD patients who were continuously followed up or referred to our hospital from January 2018 to September 2022. We further calculated the proportion of clinical score improvement: (acute‐follow‐up)/(acute‐baseline); poor prognosis was assigned to 0%–33% improvement. The relationship between treatment delay and poor prognosis was evaluated with clustered Receiver Operating Characteristic (ROC) analysis. A Generalized linear mixed model was used to analyze the related factors.

**Results:**

This study included a total of 144 episodes of myelitis attacks, of which 21.5% (31/144) resulted in poor prognosis. Based on the results of the clustered ROC analysis, it has been determined that treatment delay holds significant predictive value for poor prognosis (*p* = 0.001), with the optimal cut‐off point being 15 days. The generalized linear mixed model revealed that factors contributing to poor prognosis in NMOSD myelitis include age (OR, 1.041; CI, 1.013–1.071; *p* = 0.004) and treatment delay (OR, 1.034; CI, 1.014–1.054; *p* = 0.001).

**Conclusion:**

Our results confirm the treatment delay and age as predictors of poor prognosis in acute myelitis of NMOSD.

## Graphical abstract

This study aims to explore the related factors that contribute to poor prognosis in acute myelitis of neuromyelitis optica spectrum disorder. Our results confirm the treatment delay and age at attack as predictors of poor prognosis.

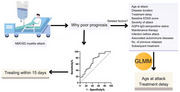

## 1 Introduction

Neuromyelitis optica spectrum disorders (NMOSD) is an immune‐mediated inflammatory demyelinating disease of the central nervous system, primarily affecting the optic nerve and spinal cord. The aquaporin‐4 (AQP4) antibodies (Abs) are a highly specific biomarker for NMOSD (Wingerchuk et al. 2015).

Approximately 68.2% of NMOSD attacks affect the spinal cord (Kleiter et al. 2016). Myelitis is a common initial symptom of NMOSD, with up to 47%–56% of patients experiencing only spinal cord involvement throughout their disease course (monophasic or recurrent myelitis) (Kitley et al. 2013; Mariano et al. 2019). The typical presentation of NMOSD myelitis is longitudinally extensive transverse myelitis (LETM) (Fadda et al. 2022). Myelitis attacks can result in paraplegia or quadriplegia, urinary and bowel dysfunction, sensory disturbances, neuropathic pain, and even life‐threatening respiratory failure, which accounts for 16.7% of NMOSD‐related deaths (Apiwattanakul et al. 2010; Du et al. 2021; Zhao‐Fleming et al. 2021; Januel et al. 2024).

NMOSD myelitis attacks are usually severe, and repeated attacks can lead to severe disability (Jarius et al. 2012). It is crucial to diagnose and treat attacks promptly to minimize permanent damage and improve patients' quality of life.

Currently, the primary therapy for myelitis attacks is high‐dose intravenous methylprednisolone pulse therapy (IVMP). Alternative therapies include intravenous immunoglobulin (IVIg), immunoadsorption (IA), and plasma exchange (PE) (Elsone et al. 2014; Trebst et al. 2014; Boedecker et al. 2022). Consensus on the treatment of myelitis attacks suggests that treatment should be initiated promptly; however, the previous studies assessing the impact of treatment delay didn't focus on myelitis attacks. Therefore, we aimed to investigate the association between treatment delay and poor prognosis in myelitis attacks, seeking to provide valuable clinical guidance.

## 2 Methods

### 2.1 Patient Selection

A retrospective cohort of 144 distinct myelitis attacks from 120 patients admitted to The Third Affiliated Hospital of Sun Yat‐sen University was enrolled in the study from January 2018 to September 2022. The inclusion criteria for subjects enrolled in the cohort were as follows: (1) patients met the clinical criteria of NMOSD from the 2015 International Panel of NMOSD diagnosis; (2) an acute attack was defined as new or worsening neurological deficits lasting for at least 24 h and occurring > 30 days after the previous attack; (3) detailed clinical status data before the attack during the clinical nadir and during a ≥ 6‐month follow‐up. All data were excluded if attacks occurred in a subintrant pattern (< 6 months) or with incomplete clinical data.

The research protocol was approved by the Ethics Committees of The Third Affiliated Hospital of Sun Yat‐sen University. Written informed consent was not required as the data were analyzed anonymously. However, verbal informed consent was obtained from the included patients.

### 2.2 Data Collection

Basic data was collected for demographic details (gender, age of attack, duration of disease, dates of disease onset), a number of relapses, comorbidities, delay of treatment initiation (the interval between symptom onset and IVMP was received, the unit of delay is days), AQP4‐Ab status, epidemiologic history (infections in the past 30 days), clinical features of spinal cord involvement (motor symptoms; sensory symptoms, bladder/bowel symptoms), spinal magnetic resonance imaging scans (All MRI scans were performed on 1.5‐T or 3‐T MRI machines and included sagittal T1 and T2 spinal cord imaging), medication data (application of IVMP, IVIg, IA, and PE in the acute phase, and use of immunosuppressants such as azathioprine, mycophenolate mofetil, methotrexate, rituximab, cyclophosphamide, and tacrolimus in the remission phase).

Severity and disability were measured before the attack, during the clinical nadir, and during a ≥ 6‐month follow‐up visit. Raters assessed patients using the Expanded Disability Status Scale (EDSS) score. Cumulative impairment was defined by ΔEDSS score = (follow‐up EDSS score) − (baseline EDSS score). Severity of the attack was defined by ΔEDSS score = (acute EDSS score) − (baseline EDSS score). We further calculated the proportion of clinical score improvement: ([acute EDSS score] − [follow‐up EDSS score])/([acute EDSS score] − [baseline EDSS score]), which provides the advantage of an interscore comparability. Poor prognosis was assigned to 0%–33% improvement.

### 2.3 Attacks Treatment

The primary treatment was methylprednisolone. Methylprednisolone was administered intravenously at a dose of 1000 mg per day for 3–5 days. The dose was reduced by half every 3 days thereafter, with the entire intravenous infusion course lasting around 12 days. Alternative therapies included five therapeutic PEs, five therapeutic IAs, or 5 days of IVIg with the intention of ameliorating an exacerbation of NMOSD. PE/IA was usually applied every other day. Patients were all recommended to start IVMP therapy except for those with contraindications. Among the patients with no or partial improvement, determined by the treating physician, acute therapy was escalated with PE, IA, or IVIg.

### 2.4 Statistical Analysis

All statistics were calculated using GraphPad Prism 9.0, R version 4.4.2, and SPSS 26.0 software. Median EDSS scores were compared by Wilcoxon paired testing with *p* < 0.05 considered statistically significant. The diagnostic efficacy of treatment delay in detecting poor prognosis was evaluated through the clustered ROC curve analysis considering intra‐patient correlation. We performed an explanatory analysis using a generalized linear mixed model (GLMM) with a random effect, considering some patients experienced multiple attacks. Collinearity and interaction between variables were checked. Fixed effects were considered significant if their *p* values were less than 0.05. Data are reported with odds ratios (ORs) and 95% confidence intervals (95% CIs).

## 3 Results

### 3.1 Characteristics of NMOSD Myelitis Attacks

Table 1 summarizes the clinical and imaging characteristics of NMOSD myelitis attacks. A total of 144 attacks of NMOSD myelitis were included in this study. The median (IQR) age at attack was 46.0 years (35.0–55.8). The median (IQR) disease duration was 4.0 (0.3–7.7) years. The median (IQR) treatment delay was 10.0 (4.3–18.8) days. The manifestation of motor symptoms occurred in 97.2%, sensory symptoms in 74.3%, and sphincter involvement in 49.3% of attacks. The median baseline EDSS score of 2.0 worsened to 4.5 at nadir (*p* < 0.001) and then improved to 2.5 at follow‐up (*p* < 0.001) after treatment. This reinforced the notion that acute treatment, on average, leads to improvement in function. Immunosuppressants were used after 131 episodes (81.0%). The results showed that 31 episodes (21.5%) had a poor prognosis.

**TABLE 1:** The clinical and imaging characteristics of NMOSD myelitis attacks.

	Cohort (*N* = 144)
Gender (female) (%)	130 (90.3%)
Age at attacks, median (IQR)	46.0 (35.0–55.8)
Disease duration (years) 1, median (IQR)	4.0 (0.3–7.7)
Preceding attacks, median (IQR) First attack (%) Second attack (%) ≥ 3 attacks (%)	2 (1–4) 27 (18.7) 22 (15.3) 95 (66.0)
Treatment delay, median (IQR)	10.0 (4.3–18.8)
Manifestations of attacks	
Sensory (%)	107 (74.3)
Motor (%)	140 (97.2)
Sphincter (%)	71 (49.3)
AQP4‐IgG‐seropositive status (%)	126 (87.5)
Infection before attack (%)	18 (12.5)
Baseline EDSS score, median (IQR)	2.0 (0.0–3.0)
Acute EDSS score, median (IQR)	4.5 (2.5–7.5)
Follow‐up EDSS score, median (IQR)	2.5 (2.0–4.0)
Lesion location	
Cervical (%)	114 (79.2)
Thoracic (%)	112 (77.8
Conus (%)	3 (2.1)
Therapy	
IVMP (%)	144 (100.0)
PE (%)	3 (2.1)
IA (%)	8 (5.6)
IVIg (%)	56 (38.9)
Maintenance therapy (%)	131 (91.0)
Severity of attack, median (IQR)	2.5 (1.0–5.0)
Complete recovery (%)	60 (41.7)
Poor prognosis (%)	31 (21.5)

Abbreviations: AQP4‐IgG, aquaporin 4; EDSS, Expanded Disability Status Scale; IA, immunoadsorption; IQR, interquartile range; IVIg, intravenous immunoglobulin; IVMP, intravenous methylprednisolone pulse therapy; NMOSD, Neuromyelitis optica spectrum disorder; PE, plasma exchange.

**FIGURE 1:**
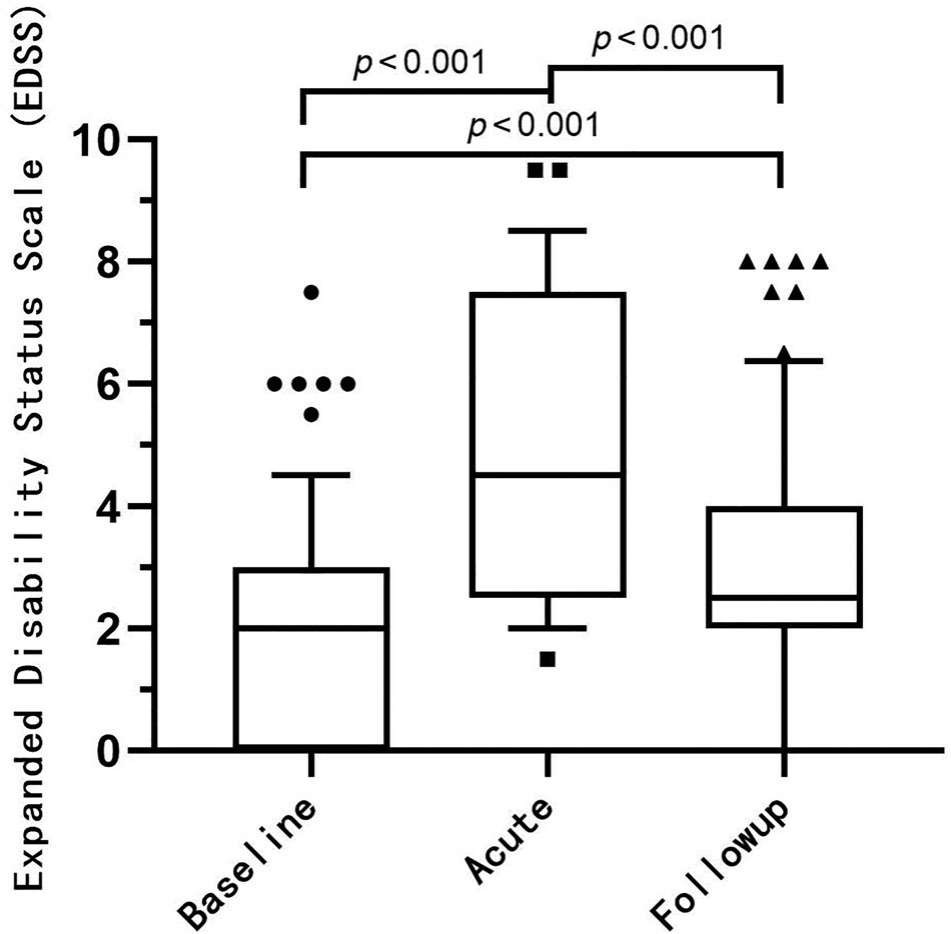
Changes in Expanded Disability Status Scale (EDSS) scores during acute NMOSD myelitis attacks. There was a significant difference in the EDSS scores at nadir compared to baseline, and there was a significant improvement at follow‐up compared to nadir. But the EDSS scores at follow‐up were significantly higher than baseline, suggesting a permanent residual disability.

As seen in Figure 1, The EDSS scores at nadir differed significantly from the scores at baseline. And there was a significant improvement of EDSS scores at follow‐up compared to nadir. However, the EDSS score at follow‐up was significantly higher than at baseline, which indicates the presence of permanent disability.

### 3.2 Treatment Delay Holds Predictive Value for Poor Prognosis

**FIGURE 2:**
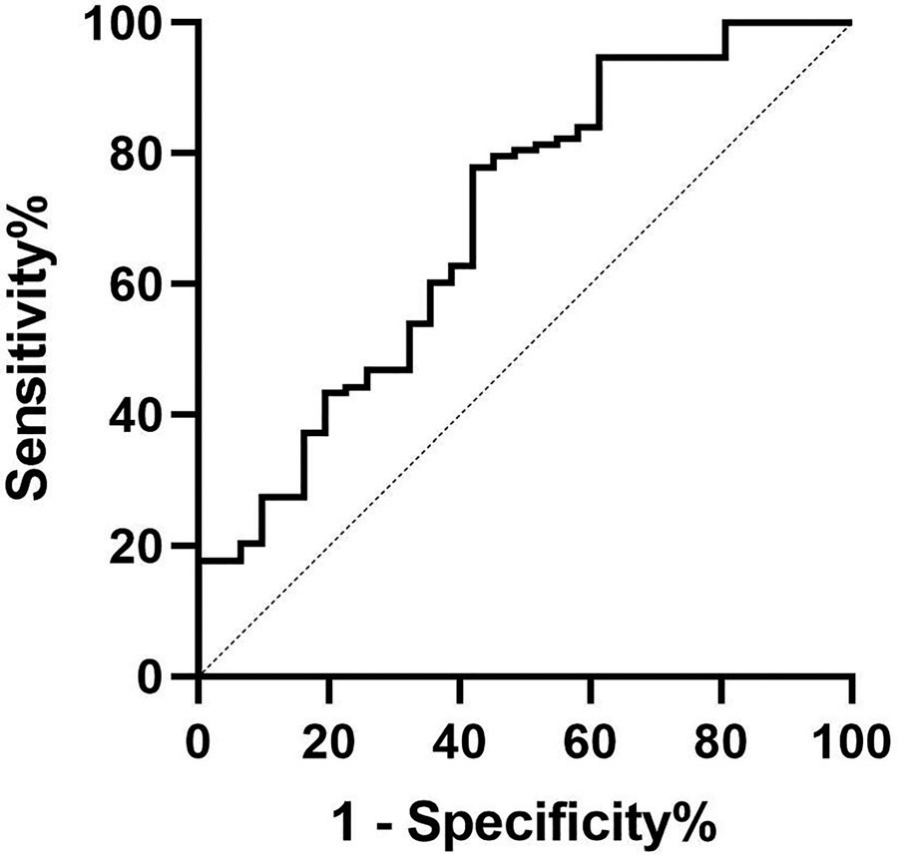
Clustered receiver operating characteristic curves of treatment delay for the prediction of poor prognosis.

Treatment delay was further tested by clustered ROC curve analysis considering intra‐patient correlation to determine its diagnostic ability and the optimal cut‐off point for the prediction of poor prognosis. The ROC curve analysis provided increased treatment delay as significant (*p* = 0.001) in identifying poor prognosis (area under the curve [AUC] = 0.697) (Figure 2). According to the ROC analysis, the optimal cut‐off values for treatment delay were determined to be 15 days for the detection of poor prognosis with a sensitivity of 58.1% and specificity of 77.9%.

### 3.3 Treatment Delay and Age at Attack Are Significantly Associated With Poor Prognosis

We performed an explanatory analysis using GLMM with a random effect, considering some patients experience multiple attacks. The results are shown in Table 2. The GLMM identified the age at attack (OR, 1.041; CI, 1.013–1.071; *p* = 0.004) and treatment delay (OR, 1.034; CI, 1.014–1.054; *p* = 0.001) as significantly associated with poor prognosis. Whereas AQP4‐IgG‐seropositive status (OR, 0.495; CI, 0.166–1.475; *p* = 0.207) demonstrated a moderate influence but was not significant. In contrast, disease duration, baseline EDSS score, the severity of the attack, maintenance therapy, infection before the attack, associated autoimmune diseases, number of previous relapses, and subsequent treatment were not predictive of poor prognosis.

**TABLE 2:** Generalized linear mixed model analyses of predictive factors of poor prognosis.

Variables	GLMM analysis OR (95%CI)	*p*
Age at attack	1.041 (1.013–1.071)	**0.004**
Disease duration	0.979 (0.915–1.048)	0.548
Treatment delay	1.034 (1.014–1.054)	**0.001**
Baseline EDSS score	0.967 (0.728–1.285)	0.819
Severity of attack	0.910 (0.757–1.094)	0.317
AQP4‐IgG‐seropositive status	0.495 (0.166–1.475)	0.207
Maintenance therapy	0.906 (0.241–3.407)	0.884
Infection before attack	1.479 (0.457–4.784)	0.513
Associated autoimmune diseases	0.505 (0.057–4.495)	0.540
No. of previous relapses	0.992 (0.856–1.149)	0.912
Subsequent treatment	1.498 (0.655–3.428)	0.339

*Note*: Bold value: Significant value (*p* < 0.05).

Abbreviations: AQP4‐IgG, aquaporin 4; EDSS, Expanded Disability Status Scale.

## 4 Discussion

In this study, we analyzed the clinical characteristics of NMOSD myelitis attacks related to the poor prognosis. We aimed to investigate the correlation between acute phase treatment duration and adverse prognosis. In our previous study, we found that myelitis attacks were the most common clinical manifestation of NMOSD attacks in South China, accounting for approximately 60% of the attacks (Wang et al. 2021). In this study, it is indicated that treatment delay is predictive of poor prognosis of myelitis attacks.

In clinical practice, we have observed that attacks of NMOSD myelitis respond more favorably to IVMP compared to optic neuritis. Although our study is retrospective, clinical scores were prospectively obtained (as EDSS scores are always prospectively collected for patients with NMOSD in our center) to ensure a reliable analysis. It is shown that 41.9% of myelitis attacks are treatable back to baseline neurological function—at least as measured by EDSS. However, the bad news is that 21.5% of these attacks result in poor prognosis. To prevent permanent residual disability, there is a need to further investigate the related factors that contribute to poor prognosis. We here report that treatment delay holds significant predictive value for poor prognosis, with the optimal cut‐off point being 15 days.

Our study demonstrates that some early‐treated patients (14.0%) still have a poor prognosis despite receiving treatment within 15 days. This suggests that there are other factors driving the poor prognosis. GLMM confirms that age at attack is one of the predictive factors of poor prognosis. It is prudent to note that older age at attack is associated with a higher probability of reaching a poor prognosis. This may be due to the impaired regulatory ability of cytokines and the immune system with altered T‐cell activity occurring during aging, leading to inadequate remyelination and irreversible axonal injury (Collongues et al. 2014). These make it relatively difficult for the elders to minimize the accumulation of disability.

There is a meaningful benefit in treating within 15 days of symptom onset, and this study supports advocacy efforts to treat myelitis attacks sooner rather than later to reduce the odds of a poor prognosis. The bad news is that long‐term outcomes from attacks are strongly correlated with age at attack, which is uncontrollable. This suggests the need for more careful disease management in elderly patients.

Several limitations of our study need to be considered while interpreting our results. First, because there is a shortage of plasma in our country, IVIg is the most common alternative therapy in our cohort. Second, although patients with NMOSD at our center were from all over China, this single‐center cohort study might not reflect the myelitis features of all Chinese patients with NMOSD totally. Further studies are needed to mitigate damage from attacks and improve long‐term outcomes in NMOSD patients.

## 5 Conclusion

Despite some limitations, this retrospective cohort study suggests that the age at attack and treatment delay are the independent predictors of poor prognosis in acute myelitis of NMOSD.

## Author Contributions

**Luyao Zhou**: writing–original draft, methodology, formal analysis, resources. **Ziyu Liao**: resources, methodology, supervision. **Yingming Long**: resources, supervision. **Zhibin Li**: supervision. **Wei Qiu**: supervision, methodology. **Zefeng Tan**: writing–review and editing, supervision, methodology. **Tingting Lu**: conceptualization, funding acquisition, supervision, project administration.

## Conflicts of Interest

The authors declare no conflicts of interest.

### Peer Review

The peer review history for this article is available at https://publons.com/publon/10.1002/brb3.70472

## Data Availability

Data available on request.
